# Off-label use of abrocitinib in dermatology: A systematic literature review with narrative summary

**DOI:** 10.1016/j.jdin.2025.11.025

**Published:** 2025-12-13

**Authors:** Zheng Su, Chen Sun, Wen-Hui Yao, Pan Wang, Yue-Ping Zeng

**Affiliations:** Department of Dermatology, State Key Laboratory of Complex Severe and Rare Diseases, Peking Union Medical College Hospital, Chinese Academy of Medical Sciences and Peking Union Medical College, National Clinical Research Center for Dermatologic and Immunologic Diseases, Beijing, China

**Keywords:** abrocitinib, JAK inhibitor, off-label, review, treatment

*To the Editor*: Abrocitinib is a selective Janus kinase 1 inhibitor that is approved for the treatment of moderate-to-severe atopic dermatitis. To date, there is emerging evidence reporting the off-label success of abrocitinib in various refractory skin diseases. This systematic review summarizes the current clinical trials and case reports concerning the off-label uses of abrocitinib. A search was conducted in the MEDLINE/PubMed, Embase, and ClinicalTrials.gov using the following terms “abrocitinib” OR “PF-04965842” (up to September 1st, 2025). The quality assessment was performed according to the guidelines provided by the Centre for Evidence-Based Medicine at Oxford.^1^ Finally, 83 published articles were included in this review (Table I and Supplementary Table I, available via Mendeley at https://data.mendeley.com/datasets/vd8y4gbymn/1). Two unpublished clinical trials of abrocitinib in patients with moderate-to-severe chronic hand eczema (NCT06283550) and sarcoidosis (NCT05696795) are in progress.

**Table I:** Summary of main findings on abrocitinib treatment for various dermatoses

Condition	Study design (sample size)	Posology	Outcome	Adverse effects	OCEBM	Author (y)
Hand eczema	Prospective real-world cohort study (*n* = 103)	200 mg once daily, 28 wk	60% patients achieved HECSI 90 at wk 16	Nausea, acneiform eruption	2	Kamphuis et al (2024)
	Prospective real-world cohort study (*n* = 12)	100 mg once daily, 16 wk	All patients reached HECSI 90 at wk 16	No	2	Li et al (2024)
Prurigo nodularis	Prospective real-world cohort study (*n* = 10)	24 wk; 100 mg once daily	NRS scores from 8-9 to 4 within 1 d, and IGA scores from 4 to 1-2 in 16 wk	Lower extremity edema (*n* = 1)	2	Wang et al (2025)
	Phase 2, open-label, nonrandomized controlled trial (*n* = 10)	12 wk; 200 mg once daily	PP-NRS scores decreased by 78.3% in PN at wk 12	Acneiform eruption (*n* = 2)	3	Kwatra et al (2024)
	Retrospective cohort study (*n* = 12)	24 wk; 100/200 mg once daily	33%, 83% and 100% patients achieved PP-NRS reduction≥4 at wk 4, wk 16, and wk 24, resp.	Dyslipidaemia, headache	3	Avallone et al (2025)
	Case series (*n* = 4)	12 wk; 200 mg once daily	PP-NRS reduced ranging from 87.5% to 100%	No	4	Demirbas et al (2025)
Plaque psoriasis	Phase 2, randomized, double-blind, placebo-controlled study (*n* = 59)	4 wk; 200 mg/400 mg once daily/200 mg twice a day	Patients achieving PASI 75 was 17% for the placebo and 200 mg OD groups, 50% for the 400 mg OD group and 60% for the 200 mg TD group at wk 4	Nausea (*n* = 8), headache (*n* = 7)	1	Schmieder et al (2018)
Alopecia areata	Retrospective cohort study (*n* = 13)	8.85 ± 4.36 mo; 50-200 mg once daily	46.15%, 53.85%, and 38.46% patients achieved SALT score≤20, SALT50, and SALT75 at least 3 mo, resp.	Folliculitis (*n* = 2), nausea (*n* = 2), laboratory abnormalities (*n* = 2)	3	Zhang et al (2025)
	Case series (*n* = 6)	12 wk; 100 mg once daily	All patients (SALT score≥50) achieved a SALT score≤20 after 8 wk	No	4	Lin et al (2025)
Granuloma annulare	Case series (*n* = 10)	6 mo; 200 mg once daily	6 patients had a significant reduction in both body surface area (mean 9.6 to 2.2) and Granuloma Annulare Severity and Morphology Instrument (mean 32.7 to 7.0)	Nausea (*n* = 2)	4	Junejo et al (2024)
	Case series (*n* = 4)	4-20 wk; 100 mg/200 mg once daily	Partial improvement in 4-16 wk	Upper respiratory infection (*n* = 1)	4	Stratman et al (2025)
Vitiligo	Prospective real-world cohort study (*n* = 11)	6 mo; 100 mg once daily × 4m 100 mg every other day × 2m	An average improvement of 14.36% and 22.07% of BSA and VASI in 6 mo	Headache (*n* = 1), dizziness (*n* = 1), gastrointestinal discomfort/nausea (*n* = 1)	2	Xu et al (2024)

*BSA*, Body surface area; *HECSI*, Hand Eczema Severity Index; *OCEBM*, Centre for Evidence-Based Medicine at Oxford; *PASI*, Psoriasis Area Severity Index; *PP-NRS*, Peak Pruritus Numerical Rating Scale; *SALT*, Severity of Alopecia Tool; *VASI*, Vitiligo Area Severity Index.

Abrocitinib has shown good therapeutic efficacy in refractory eczema (such as hand eczema), prurigo nodularis, and chronic pruritus skin diseases. A phase 3 trial has suggested abrocitinib was more efficacious than dupilumab in adults with moderate-to-severe atopic dermatitis in inducing early reductions of itch and atopic dermatitis disease signs.^2^ Therefore, for these patients with poor response to dupilumab, abrocitinib seems to be a promising alternative. Additionally, abrocitinib may alleviate paradoxical reactions induced by monoclonal antibodies (such as dupilumab-associated head and neck erythema, psoriasiform dermatitis, and interleukin-17/tumor necrosis factor-α inhibitors induced eczematous-like eruptions) without distinct side effects, as an optional solution in clinical practice.

Abrocitinib tended to improve psoriasis. A phase II study with 59 patients has been investigated for moderate-to-severe plaque psoriasis. However, at week 4, only 17% of patients in the placebo and 200 mg once daily groups achieved Psoriasis Area Severity Index 75 response, versus 50% with 400 mg once daily and 60% with 200 mg twice daily,^3^ suggesting the limited utility of abrocitinib for treating psoriasis.

Abrocitinib was effective in promoting hair regrowth in both atopic and non-atopic alopecia areata,^4^ with initial responses observed as early as 4 months in all reported cases. Additionally, a prospective observational study and 3 case reports described successful improvement in vitiligo to varying degrees with oral abrocitinib.

Notably, current literature showed remarkable effectiveness of abrocitinib against refractory granulomatous dermatoses, with the majority of patients achieving near-complete resolution of lesions within several weeks. A prospective, open-label case series with abrocitinib in generalized granuloma annulare has showed that a marked reduction in both body surface area in all 6 patients.^5^

Successful treatment of various other dermatoses with abrocitinib was summarized in Supplementary Table I, available via Mendeley at https://data.mendeley.com/datasets/vd8y4gbymn/1. However, caution is warranted as the data largely consist of low-level evidence from uncontrolled reports with considerable selection bias. In conclusion, although limited by a small sample size, the data collected until now on the use of abrocitinib in dermatology is encouraging. Further large-scale studies and long-term surveillance are necessary to evaluate the efficacy and safety of abrocitinib in various dermatoses.

## Conflicts of interest

None disclosed.
